# Pharmacokinetics of Drugs in Cachectic Patients: A Systematic Review

**DOI:** 10.1371/journal.pone.0079603

**Published:** 2013-11-08

**Authors:** Katja Trobec, Mojca Kerec Kos, Stephan von Haehling, Jochen Springer, Stefan D. Anker, Mitja Lainscak

**Affiliations:** 1 Pharmacy Department, University Clinic of Respiratory and Allergic Diseases Golnik, Golnik, Slovenia; 2 Faculty of Pharmacy, University of Ljubljana, Ljubljana, Slovenia; 3 Applied Cachexia Research, Department of Cardiology, Charité Medical School, Campus Virchow-Klinikum, Berlin, Germany; 4 Center for Cardiovascular Research, Charité Medical School, Campus Virchow-Klinikum, Berlin, Germany; 5 Norwich Medical School, University of East Anglia, Norwich, United Kingdom; 6 Center for Clinical and Basic Research, IRCCS San Raffaele, Rome, Italy; 7 Division of Cardiology, University Clinic of Respiratory and Allergic Diseases Golnik, Golnik, Slovenia; Heidelberg University, Germany

## Abstract

Cachexia is a weight-loss process caused by an underlying chronic disease such as cancer, chronic heart failure, chronic obstructive pulmonary disease, or rheumatoid arthritis. It leads to changes in body structure and function that may influence the pharmacokinetics of drugs. Changes in gut function and decreased subcutaneous tissue may influence the absorption of orally and transdermally applied drugs. Altered body composition and plasma protein concentration may affect drug distribution. Changes in the expression and function of metabolic enzymes could influence the metabolism of drugs, and their renal excretion could be affected by possible reduction in kidney function. Because no general guidelines exist for drug dose adjustments in cachectic patients, we conducted a systematic search to identify articles that investigated the pharmacokinetics of drugs in cachectic patients.

## Introduction

The pharmacokinetics of many drugs is primarily tested and thoroughly evaluated in healthy volunteers, even though this is not the target population for clinical drug use [1,2]. Chronic diseases such as cancer, chronic heart failure (HF), chronic obstructive pulmonary disease (COPD), and rheumatoid arthritis (RA) can change the pharmacokinetics of drugs, leading to possible alterations of their effects. With the progression of a chronic disease, body wasting and cachexia may develop, which induces additional changes in drug pharmacokinetics.

Cachexia is a weight-loss syndrome caused by an underlying chronic disease. The definition of cachexia has only recently been proposed as involuntary weight loss of 5% or more (or, alternatively, a body mass index (BMI) of less than 20), accompanied by at least three cofactors: decreased muscle strength, fatigue, anorexia, low fat-free mass, and/or abnormal biochemistry (increased inflammatory markers, anemia, and low serum albumin) [3,4]. The exact mechanisms of this syndrome are not yet known. Weight loss is due to whole-body wasting, including muscle and fat tissue wasting, caused by altered metabolism of fat, carbohydrates, and proteins. Chronic inflammation appears to play an important role in inducing these changes and in disrupting appetite modulation [5,6].

**Figure 1:**
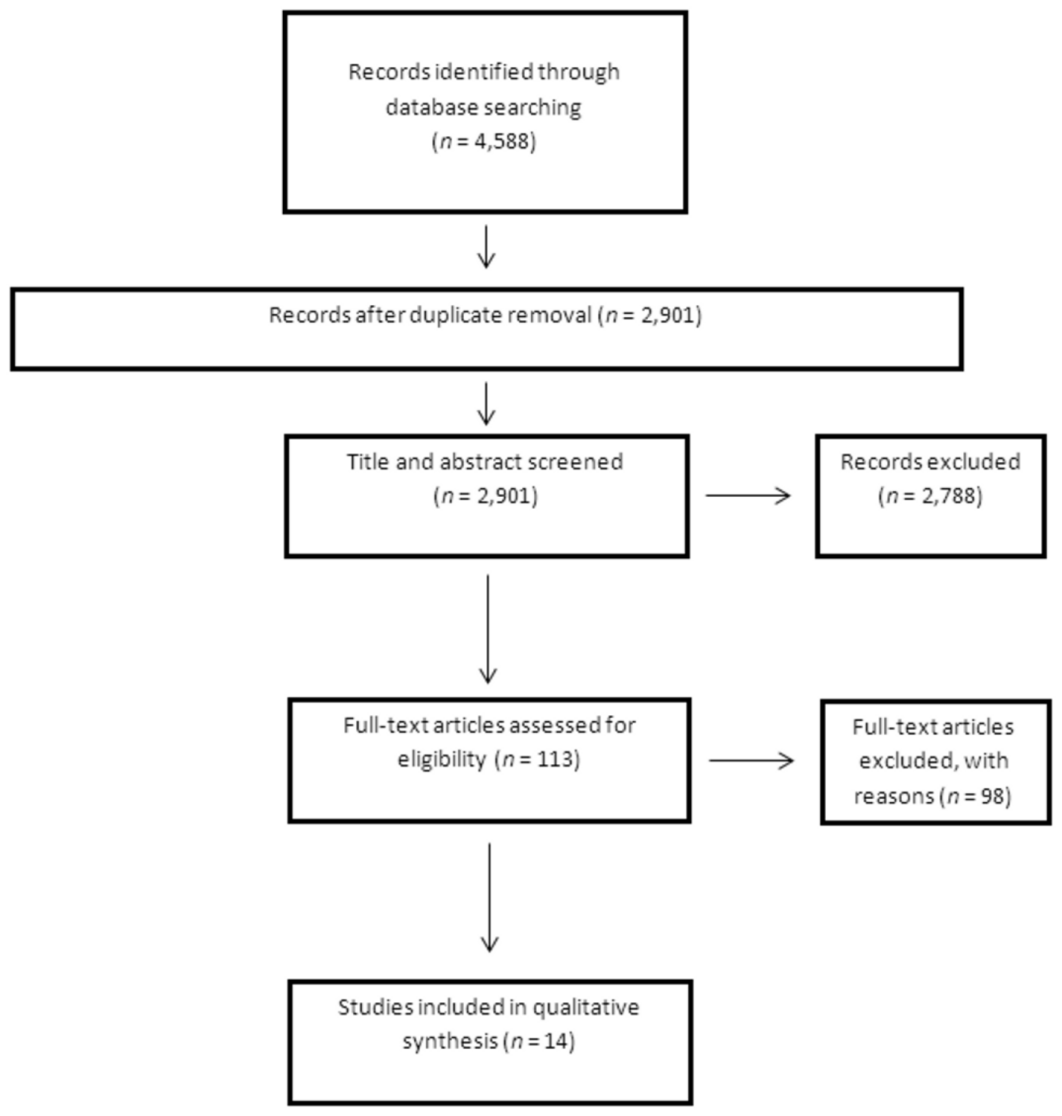
PRISMA flow diagram.

Cachexia-induced changes in metabolism, signaling pathways, and body composition may alter the pharmacokinetics of various drugs. An orally administered drug must be absorbed (A) into systemic circulation and distributed (D) throughout the body. Afterwards, formation of active and inactive metabolites (M) may occur, or the intact drug may be excreted (E) [7]. Each of these so-called ADME processes may be influenced by cachexia (Figure 1), leading to an altered concentration of the drug at the site of action, altered efficacy, or increased risk of adverse drug reactions. If the pharmacokinetics of a drug is affected by cachexia, it seems appropriate to adjust drug doses in cachectic patients, yet no general guidelines exist. In view of clinical relevance and data scarcity, we conducted a systematic literature review to identify studies that evaluated the pharmacokinetics of drugs in cachectic patients.

## Methods

### Search strategy

A systematic electronic literature search of PubMed (1950-March 2012), EMBASE (1974–2012 Week 09), the Cochrane Central Register of Controlled Trials and CINAHL with full text (1981-March 2012) was conducted according to the PRISMA statement [8]. In PubMed, the following limits were applied: English, Human, and Title/Abstract. Search terms describing body composition and pharmacokinetics were combined with terms defining several chronic diseases (Table 1). An additional search was performed in same four databases with the same limitations as previously, but with only two terms: “pharmacokinetic” AND “cachexia”. This search helped to identify the papers with clear importance for our study which could had been missed due to lack of appropriate mention of chronic disease in the title or abstract. All identified papers were assessed independently by two reviewers (KT and ML) and disagreements about their eligibility were resolved by discussion with a third reviewer (MKK).

**Table 1:** Search terms in Pubmed.

((weight loss) OR cachexia OR (body composition) OR malnutrition OR (body wasting) OR (muscle wasting) OR (fat wasting) OR (fat free mass) OR dexa OR (dual energy x ray absorptiometry) OR dxa OR (bioimpedance analysis))
AND
((pharmacokinetic or pharmacokinetics) OR (area under curve) OR (half-life) OR cmax OR tmax OR metabolism OR clearance OR elimination OR distribution OR absorption OR dosage)
AND
((chronic heart failure) OR (heart failure) OR cancer OR malignancy OR (chronic obstructive pulmonary disease) OR COPD OR (chronic kidney disease) OR CKD OR (rheumatoid arthritis) OR HIV OR AIDS OR (human immunodeficiency virus) OR cirrhosis)

COPD = chronic obstructive pulmonary disease, CKD = chronic kidney disease, HIV = human immunodeficiency virus, AIDS = acquired immune deficiency syndrome

### Inclusion criteria

We included studies performed in patients with one of the following chronic diseases: chronic HF, COPD, cancer, chronic kidney disease (CKD), rheumatoid arthritis, AIDS (acquired immunodeficiency syndrome), or cirrhosis. We searched for two types of interventions:

- Measurement of drug concentrations in biological samples in order to assess pharmacokinetic parameters; and
- Body-composition or weight-loss assessment, or diagnosis of cachexia

The studies included compared pharmacokinetic parameters in patients with chronic disease and cachexia (or wasting or altered body composition) with pharmacokinetic parameters in healthy people, or in patients with the same chronic disease but without cachexia. Studies that correlated the parameters of body composition with the pharmacokinetics of drugs in patients with chronic disease were also included.

### Exclusion criteria

Papers were excluded if they were in a language other than English and if studies were not conducted in humans. Because we were focused only on drug pharmacokinetics, we excluded studies considering intestinal absorption of sugars, studies of labeled carbohydrates and amino-acid metabolism, and studies that investigated the application of substances naturally occurring in the human body (e.g., hormones, amino acids, etc.).

### Studies included in the review and data synthesis

**Figure 2:**
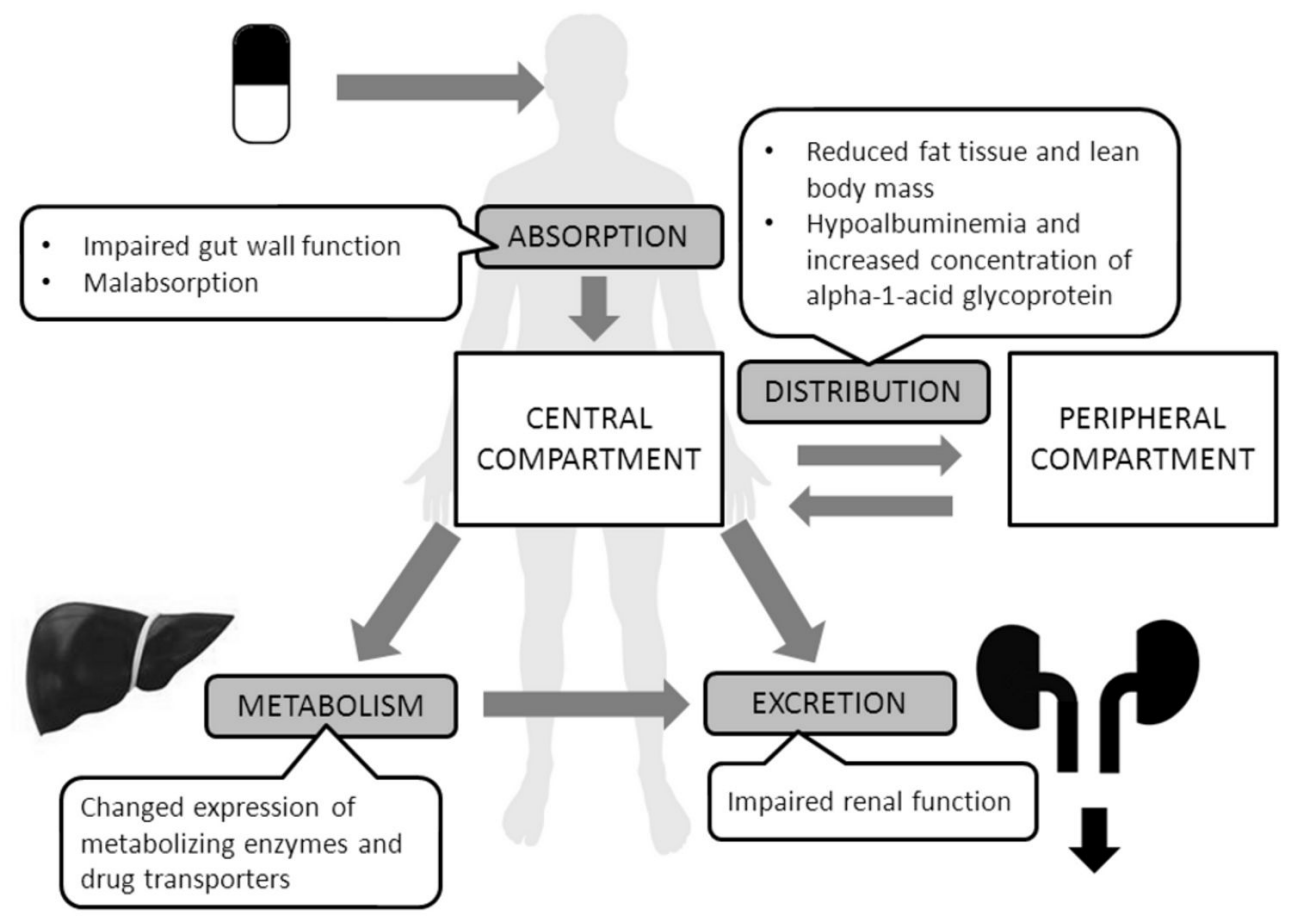
ADME processes in cachexia.

The flow-chart diagram in Figure 2 shows the total number of papers screened and number of papers that met inclusion criteria. Data about the drugs investigated, patients’ characteristics, diagnosis of cachexia, and pharmacokinetics were extracted from the studies, as was the relative comparison of pharmacokinetic parameters between cachectic and non-cachectic patients.

## Results

The systematic search identified 4,588 papers (Figure 1). After the removal of the duplicates, the titles and abstracts of 2,901 records were screened. The vast majority of records (2,788) were excluded due to evident absence of topic of interest and 113 full-text papers were assessed for eligibility. Finally, 14 papers were included in the analysis. Five of these were designed as studies that compared pharmacokinetic parameters in non-cachectic patients versus cachectic patients [9–13]. The results are presented in Table 2. Four studies included patients with wasting and concomitant diarrhea [14–17] and are represented separately (Table 3). The remaining five studies [18–22] correlated pharmacokinetic parameters in patients with chronic disease with parameters of body composition (Table 4).

**Table 2:** Studies comparing pharmacokinetic parameters in cachectic patients versus non-cachectic patients [9-13].

**Study**	**Naito et al., 2012**	**Heiskanen et al., 2009**	**Herrington et al., 2006**	**Gatti et al., 1999**	**Pollock et al., 2009**
**Drug**	OXYCODONE	FENTANYL	CARBOPLATIN	RIFABUTIN	NEVIRAPINE
**Drug application**	Oral (extended-release tablets)	Transdermal patch	Intravenous infusion	Oral	Oral
**Dosing**	Titration of the dose	/	/	Single dose	Multiple doses, steady state
**Patients (disease)**	Cancer patients	Cancer patients	Patients scheduled to receive a carboplatin 1 h infusion	HIV-infected patients	HIV-infected children
**Patients (*n*)**	47	20	28	20	37
**Average age of**	66	64 (normal weight)	Median age:	37 (without wasting syndrome)	4.4
**patients (years)**		62 (cachectic)	61 (BMI ≥ 27), 67 (cachexia)	35 (with wasting syndrome)	
**Gender (% male)**	70%	45%	57%	80%	57%
**Definition of cachexia**	Glasgow prognostic score (GPS):	BMI < 18 kg/m^2^	Serum creatinine < 70.7 μM	Weight loss > 10% of usual weight	Nutritional status according to
	CRP > 1.0 mg/dL = 1 score		≥ 5% weight loss over 6-month	during the year preceding the study	weight for height (wt/ht):
	Albumin < 3.5 g/dL = 1 score		period		Normal weight:
			Serum albumin < 34 g/L		wt/ht > 85% of median
			BMI < 27		Mild to moderate malnutrition:
					70< wt/ht < 85%
**Patient groups**	GPS = 0 (*n* = 7)	Normal weight (*n* = 10)	BMI≥ 27 (*n* = 19)	No wasting syndrome (*n* = 10)	Normal (*n* = 25)
	GPS = 1 (*n* = 21)	Cachectic patients (*n* = 10)	Cachectic patients (*n* = 9)	Wasting syndrome (*n* = 10)	Malnourished (*n* = 12)
	GPS = 2 (*n* = 19)				
**Measured drug concentrations**	Plasma concentration (in ng/mL per mg/kg) of oxycodone and its metabolite noroxycodone 12 h after the evening dose.	Plasma fentanyl concentrations divided by the dose at baseline, and 4, 24, 48, and 72 h after patch application.	Plasma concentrations of carboplatin 0.5, 1, 1.5, and 6 h after the beginning of infusion.	Plasma concentrations of rifabutin at predose and 0.5, 1, 2, 3, 4, 6, 8, 24, 48, 72, and 96 h post dose.	Plasma concentrations of total and unbound nevirapine at pre-dose, 2, 4, 8, and 12 h post dose.
**Pharmacokinetic model**	None	None	One-compartment model	Non-compartmental analysis	Non-compartmental analysis
**Major findings**	Higher concentrations of oxycodone in cachectic patients	Higher doses of fentanyl required in cachectic patients	No difference in pharmacokinetic parameters (t_½_, Cl, V_d_) between the two groups	c_max_ and c_24h_ significantly higher in patients with wasting syndrome	No effect of malnutrition on total nevirapine AUC_0–12_, c_max_, and c_trough_
	No difference in noroxycodone concentrations	Fentanyl plasma concentrations at 48 and 72 h post dose significantly lower in cachectic patients		AUC and CL/F normalized to body weight were similar between groups	Trend to lower total nevirapine c_max_, and AUC_0–12_ in malnourished vs. normal children (NS), related to dose/m² rather to malnutrition per se
	Decreased hepatic conversion of oxycodone to noroxycodone with CYP3A4	The difference at 4 and 24 h post dose was not significant		Trend to smaller V_d_/F and shorter t_½_ (NS) in patients with wasting syndrome	No differences in unbound fraction of nevirapine
	GPS affected the incidence of dose escalation (OR = 0.268) and central adverse reaction (OR = 4.24)				No differences in unbound nevirapine AUC_0–12_

c_max_ = maximal concentration, c_trough_ = minimal concentration, c_24h_ = concentration at 24 h post dose, AUC = area under concentration time curve, AUC_0–12_ = area under concentration time curve in first 12 h after drug application, t_½_ = half-life, F = fraction of absorption, V_d_ = volume of distribution, CL = clearance, CRP = C-reactive protein, HIV = human immunodeficiency virus, CYP = cytochrome, BMI = body mass index, OR = odds ratio, NS = not statistically significant

**Table 3:** Pharmacokinetic studies in patients with wasting and concomitant diarrhea [14-17].

**Study**	**Mouly et al., 2001**	**Trout et al., 2004**	**Brantley et al., 2003**
**Drug**	GANCICLOVIR	SAQUINAVIR	STAVUDINE, ZIDOVUDINE, DIDANOSINE, LAMIVUDINE
**Drug application**	Oral (hard-gelatin capsules)	Oral (hard-gelatin capsules)	/
**Dosing**	Single oral dose	Single oral dose, ingested with grapefruit juice (CYP3A4 inhibition for higher saquinavir bioavailability)	/
**Patients (disease)**	HIV-infected patients	HIV-1-infected patients	HIV-infected patients together with at least one AIDS-defining illness
**Patients (*n*)**	42	100	19
**Average age (years)**	37 (Group A), 36 (Group B), 39 (Group C)	40 (Group 1), 40 (Group 2), 39 (Group 3)	29 (diarrhea/ wasting), 31 (outpatient)
**Gender (% male)**	79%	81%	68%
**Definition of cachexia**	Loss of ≥ 10% of body weight from baseline weight during the last year of follow-up	Loss of > 10% body weight during the past month	Weight loss > 10% below baseline during 2 months prior to entry
**Definition of diarrhea**	More than three loose bowel movements a day for at least 4 weeks	More than three daily bowel movements for at least 3 weeks and not related to antiretroviral therapy	Three or more stools with decreased consistency during at least 8 of the 10 days prior to enrollment or
			Intermittent diarrhea for 2 weeks over the 2 months prior to entry
**Patient groups**	Group A: HIV-infected patients without AIDS defining illness (stage A or B) (*n* = 15)	Group 1: asymptomatic patients (*n* = 30)	Patients with diarrhea and wasting (*n* = 12)
	Group B: AIDS patients (stage C) without diarrhea and weight loss (*n* = 13)	Group 2: AIDS symptomatic patients without weight loss or diarrhea (*n* = 37)	Outpatients with a history of a serological HIV test and an AIDS-defining illness without diarrhea or weight loss in the preceding 2 months (*n* = 7)
	Group C: AIDS patients (stage C) with diarrhea and/or weight loss (*n* = 14)	Group 3: AIDS symptomatic patients with severe body weight loss and/or diarrhea (*n* = 33)	
**Measured drug concentrations**	Ganciclovir serum concentrations at 0.5, 2, 4, 8, 12, and 24 h post dose	Saquinavir plasma concentrations in three time points (one sample in each time period: 0 to 1.5 h, 2 to 4 h, and 5 to 12 h post dose)	Plasma stavudine and zidovudine concentrations 35–45 min post dose. Didanosine plasma concentration 45–65 min post dose. Lamivudine 55–95 min post dose.
**Pharmacokinetic (PK) model**	Two-compartment model with first-order absorption.	One-compartment model with first-order elimination and first-order	None.
	**Mouly et al., 2000**: Non-compartmental analysis.	absorption with a lag time.	
**Findings**	Group A and B had nearly super-imposable concentration-time profiles, while in Group C the profile reached higher concentrations. Lower systemic clearance (CL/F) in Group C.	Significant difference between CL/F, V/F, k_a_, T_lag_, and k_el_ among groups (highest in Group 1, lowest in Group 3) and AUC (lowest in Group 1, highest in Group 3).	Stavudine and didanosine plasma concentrations lower in patients with diarrhea (no statistical test applied).
	Lower central volume of distribution (V1/F) in Group C.	C_max_ significantly higher in Group 3 compared to Group 1.	
	**Mouly et al., 2000:**	t_max_ significantly shorter in Group 3 compared to Group 1.	
	Higher C_max_, AUC_0–24h_, and AUC_0–∞_ in Group C versus Group A+B.		
	Lower Cl/F in Group C.		
	No difference in t_½_ and t _max_.		
**Conclusion**	Patients with weight loss and diarrhea had reduced apparent oral clearance (Cl/F) by approximately 50%; higher intestinal permeability is suggested to be the cause.	Increase in AUC in Group C is due to the decreased PGP efflux of saquinavir in intestinal wall and increased dose of saquinavir expressed in mg per kg (due to decreased total body weight).	
		Increased intestinal permeability in Group C (proven by sugar absorption test) could explain increased paracellular transport of saquinavir.	

c_max_ = maximal concentration, AUC_0–24h_ = area under concentration time curve in first 24 h after drug application, AUC_0- ∞_ = area under concentration time curve from time of drug application to infinite time, t_½_ = half- life, t_max_ = time of maximal concentration, T_lag_ = lag time, k_a_ = absorption rate constant, k_el_ = elimination rate constant, V1/F = central volume of distribution divided by bioavailability, V/F = volume of distribution divided by bioavailability, CL/F = clearance divided by bioavailability, HIV = human immunodeficiency virus, AIDS = acquired immune deficiency syndrome, CYP = cytochrome, PGP = P-glycoprotein

**Table 4:** Pharmacokinetic studies in patients with determined body composition [18-22].

**Study**	**Gatti et al., 1998**	**Prado et al., 2011**	**Gusella et al., 2002**	**Kumar et al., 1987**	Kuester et al., 2009*
**Drug**	RIFABUTIN	EPIRUBICIN	FLUOROURACIL	METHOTREXATE	MATUZUMAB
**Drug application**	Oral (tablets)	Intravenous infusion (median duration 20 min)	Intravenous bolus injection (2 min)	Intravenous bolus injection	Multiple 1 h intravenous infusions
**Dosing**	Different regimens, steady state	100 mg/m^2^ BSA,every 3 weeks	425 mg/m^2^ daily for 5 days (six consecutive cycles). Study performed on second day of the first therapy cycle.	50 mg/m^2^ BSA	Various dosing regimens**
**Patients (disease)**	HIV-infected patients.	Breast cancer patients.	Colorectal cancer patients.	Children with malignancies who were “not obviously cachectic”	Various types of advanced carcinoma
**Patients (*n*)**	30	24	34	6	90
**Average age (years)**	34	53	66	Range: 1–15	Median age: 60
**Gender (% male)**	70%	0%	38%	67%	59%
**Parameters of body composition**	Cachexia index = (1 − actual patient weight/ ideal body weight)	CT images analysis: (muscle cross-sectional area, muscle attenuation, estimated total lean body mass, fat cross-sectional area, estimated total body fat mass)	Body composition measured by BIA	Nutritional anthropometry (height, weight, head/arm/chest/muscle circumference, subscapular/triceps skinfold thickness), relative weight	FFM calculated from body weight and BMI
**Measured drug concentrations**	Rifabutin plasma concentration at time 0, and once within the following intervals: 0–4, 4–12, 12–24, 24–48, and 48–96 h post dose	Epirubicin plasma concentrations 1 and 24 h after the end of epirubicin infusion.	Fluorouracil plasma concentrations at 0, 2.5, 5, 10, 15, 20, 30, 45, and 60 min after drug administration.	Methotrexate plasma concentration at various time intervals from 30 min to 24 h after drug administration	Matuzumab serum concentrations pre- and post-infusions**
**Pharmacokinetic (PK) model**	Two-compartment open model with first-order rate constants for absorption and elimination	One-compartment model, three-compartment model, non-compartmental analysis	One- and two-compartment model	Two-compartment model	Two-compartment model
**Findings**	Cachexia index did not significantly influence Cl/F or V_p_/F.	None of the covariates were significant in the one-compartment model approach.	Significant but poor correlations between:	Relative weight highly negatively correlated with elimination half-life.	FFM influenced linear clearance.
	Cachexia index > 35 resulted in a significant decrease in V_p_/F.	Significant correlation between log-clearance and LBM.	Cl and BW, BSA, TBW, FFM, BCM	No significant correlation between relative weight and volume of central or tissue compartment.	
			V_ss_ and BW, TBW, FFM.		
			Higher r^2^ if correlations were performed for males and females separately.		
			Multiple regression:		
			Cl sig. correlated with sex and FFM,		
			V_ss_ sig. correlated with sex and TBW.		

AUC = area under concentration time curve, F = fraction of absorption, V_ss_ = distribution volume at steady state, V_p_ = volume of peripheral compartment, Cl = clearance, DEXA = dual-energy X-ray absorptiometry, CT = computed tomography, BW = body weight, TBW = total body water, FFM = fat-free mass, BSA = body surface area, sig. = significantly

* Data taken from development dataset.

** Exact data is available in the paper by Kuester et al. (21)

The studies included were performed only in patients with cancer and HIV (human immunodeficiency virus). No studies described the pharmacokinetics of drugs in cachectic patients with chronic HF, COPD, RA, CKD, or cirrhosis. Sample sizes were relatively small and the definition of cachexia or wasting differed considerably. The drugs studied were antiviral drugs [13–17], cytostatics [11,19–21], tuberculostatics [12,18], and opioids [9,10], which were administered orally, intravenously, or, in one case, transdermally. One study observed the pharmacokinetics of the monoclonal antibody matuzumab [22].

The studies used different approaches to determine pharmacokinetic parameters. In some studies, only plasma concentrations were measured, whereas others applied compartmental or non-compartmental pharmacokinetic analysis.

Table 5 presents the chemical and pharmacokinetic proprieties of the drugs that were investigated in the studies cited in Tables 2 and 3. The first part of the table presents the pharmacokinetic proprieties of drugs for healthy persons, followed by the observed changes of these parameters in cachectic patients [23–29]. We only present drugs from Tables 2 and 3 because only these studies provided a relative comparison of pharmacokinetic parameters between cachectic and non-cachectic patients.

**Table 5:** Chemical and pharmacokinetic proprieties of drugs in non-cachectic population [23-29] and observed changes in cachectic patients [9-18].

**PHARMACOKINETICS IN WASTING/CACHEXIA**						**PHARMACOKINETICS IN WASTING/CACHEXIA AND DIARRHEA**			
Study	**Naito et al., 2012**	**Pollock et al., 2009**	**Gatti et al., 1999; Gatti et al., 1998**	**Heiskanen et al., 2009**	**Herrington et al., 2006**	**Mouly et al., 2000 and 2001**	**Trout et al., 2004**	**Brantley et al., 2003**	**Brantley et al., 2003**
Name of the drug	OXYCODONE	NEVIRAPINE	RIFABUTIN	FENTANYL	CARBOPLATIN	GANCICLOVIR	SAQUINAVIR	STAVUDINE	DIDANOSINE
Route of application	Oral	Oral	Oral	Transdermal	Intravenous	Oral	Oral	Oral	Oral
Chemical proprieties									
logP	0.3 (hydrophilic)	2.5 (lipophilic)	4.1 (lipophilic)	3.9 (lipophilic)	3.2 (lipophilic)	−1.7 (hydrophilic)	3.8 (lipophilic)	−0.8 (hydrophilic)	−0.2 (hydrophilic)
Pharmacokinetic proprieties in healthy population									
Bioavailability	60–87%	93%	20%	92% (transdermal)	/	5%	4%	86.4%	30–40%
V_d_ (L or L/kg)	2.6 L/kg	1.21 L/kg	9.32 L/kg	4–6 L/kg	16 L	0.74 L/kg	700 L	46 L	1.08 L/kg
Plasma protein binding	45%	60%	85%	80–85%	Very low	1–2%	98%	Negligible	< 5%
Enzyme systems of hepatic clearance	CYP3A4 (major)	CYP3A4 (major)	CYP3A4 (major)	CYP3A4	/	/	CYP3A4 (major)	Phosphorylation	Unknown
	CYP2D6 (minor)	CYP2B6 (minor)	CYP1A2 (minor)				CYP2D6 (minor)		
	Glucuronidation of metabolites	CYP2D6 (minor)					PGP		
		Glucuronidation of metabolites							
Fraction of drug secreted unchanged in urine	~ 19%	< 3%	53%	7–10%	70%	80–99%	1–3%	70%	55%
Other paths of excretion	/	~10% in feces	30% in feces	~ 9% in feces	/	/	81–88% in feces	3% in feces	/
Clearance or oral clearance	800 mL/min	27.5 mL/min	11.5 mL/min/kg	450–1,250 mL/min	73.3 mL/min	3.64 ± 1.86 mL/min/kg	19 mL/min/kg	406 mL/min	800 mL/min
Half-life (h)	4–5	45 (initial)	45	17 (transdermal patch)	2.6–5.9	1.7–5.8	7	0.8–1.5	1.5
		25–30 (after autoinduction)							
Change of pharmacokinetic proprieties in cachectic patients									
Plasma drug concentrations	↑	=	↑	↓	=	↑	↑	↓	↓
Plasma metabolite concentrations	=								
V_d_ or V_d_/F			↓ (due to ↓ V_p_)		=	↓ (due to ↓ V_c_)	↓		
Cl or CL/F			=		=	↓	↓		
Half-life			↓			=	↓		
AUC		=	=			↑	↑		

AUC = area under plasma concentration time curve, V_c_ = volume of central compartment, V_p_ = volume of peripheral compartment, V_d_ = volume of distribution, Vd/F = volume of distribution divided by bioavailability, Cl = clearance, CL/F = clearance divided by bioavailability, CYP = cytochrome, logP = logarithm of partition coefficient

## Discussion

Only 14 papers were selected for our final analysis, which demonstrates the lack of pharmacokinetic data in patients with cachexia or wasting. The same was observed for obese patients because both populations are generally excluded from drug-registration studies [30]. Moreover, the selected studies were conducted only in patients with cancer and HIV, although cachexia may develop in a variety of chronic diseases. This may be due to the greater prevalence of cachexia in patients with cancer or HIV [31,32] or due to the greater interest of stakeholders associated with particular diseases.

We did not include papers that investigated drug pharmacokinetics in the malnourished population without chronic disease because the presence of chronic disease is a main criterion that underlies cachexia. In addition, these publications have already been systematically reviewed [33,34]. There are also many papers that have correlated body composition parameters with pharmacokinetic parameters, although not necessarily in patients with chronic disease. Recently, a systematic review and meta-analysis of these papers was performed by McLeay et al. [35]. Our search provided five papers that established this correlation in patients with chronic diseases [18–22]. Although this was not the main scope of our article, we still find it appropriate to present papers of this kind that were identified by our search (Table 4).

This review focuses on patients with cachexia; we discuss mechanisms by which cachexia may influence ADME processes (Figure 1) and whether the findings of this systematic review fit the proposed mechanisms.

### Drug absorption in cachexia

In body wasting and cachexia, gut wall function is modified, which may alter the absorption of orally administered drugs. Changes in the gut wall are associated with weight loss regardless of the underlying chronic disease [36]. When compared to controls, patients with chronic HF may have increased bowel wall thickness, increased intestinal permeability, and impaired function of transport proteins. However, among patients with chronic HF of the same functional class, the collagen content of the mucosal wall was higher in patients with cardiac cachexia [37–39]. In addition, cardiac cachexia was associated with fat malabsorption [40] and is likely to cause bacterial translocation [41]. The effects of these changes on drug absorption are difficult to predict. The studies described in Table 3 were performed in HIV-infected patients with wasting and concomitant diarrhea. Diarrhea alone can influence the absorption of drugs differently. On the one hand, it can lead to higher elimination from the gastrointestinal tract and lower the fraction of drug absorbed, or, on the other hand, it can lead to higher intestinal permeability and higher absorption of drugs due to damage to the intestinal mucosa. Patients in studies by Mouly [15] and Trout [16] had higher AUC (area under concentration-time curve) of ganciclovir and saquinavir and lower CL/F (oral clearance), which is explained as the consequence of higher intestinal absorption. The opposite effect (lower drug concentrations in patients with diarrhea and wasting) was observed for stavudine and didanosine [17]. It seems that drugs with low bioavailability (ganciclovir, saquinavir) have an increased absorption in diarrhea due to higher intestinal permeability. On the other hand, drugs with otherwise good intestinal absorption (in our case, stavudine and didanosine) are more influenced by faster elimination of the drug from the intestinal tract, which results in lower bioavailability. From the clinical standpoint, doses of low bioavailability drugs should be lowered and doses of drugs with high bioavailability should be increased in cachectic patients with wasting and diarrhea. 

The absorption process may also take place from subcutaneous tissue when administering subcutaneous injections or transdermal patches. Because less subcutaneous fat is present in cachectic patients [42], this may affect the kinetics of the drug entering systemic circulation. Indeed, Heiskanen et al. [10] showed lower plasma concentrations of transdermal fentanyl and impaired absorption of the drug was suggested in cachectic patients. 

In summary, no study evaluated absorption process in cachexia directly. In patients with wasting and concomitant diarrhea, drugs with otherwise low bioavailability seem to have higher absorption fraction, while absorption of drugs with high bioavailability seems to be reduced. Due to changes in body composition, it is difficult to predict the absorption of subcutaneously and transdermally administered drugs in cachectic patients and alternative routes may be preferred. Nonetheless, as most of drugs for chronic disease are administered orally and in view of cumulating evidence of altered absorption in chronic disease with or without cachexia, studies focusing on drug absorption in cachexia are warranted.

### Drug distribution in cachexia

In cachectic patients, body composition differs from the normal population, which was shown by Fearon et al. in 1990 [43]. In this study, total body fat was reduced by 80% and muscle protein by 75% in cachectic cancer patients compared to controls, but there was no difference in non-muscle protein mass. Lean body mass (body protein, water, and mineral content) was reduced by 13%. Intracellular water was lower in cachectic patients, but there was no difference in the mass of extracellular water. In patients with cardiac cachexia, reduced fat mass and a trend toward lower fat-free mass was found when compared to non-cachectic CHF patients [44]. In addition to body composition changes, hypoalbuminemia may occur in advanced cachexia, causing water retention [45,46].

These changes may influence the distribution of drugs differently, depending on their lipophilicity and affinity for different tissues. A reduced mass of adipose tissue accumulates lower amounts of lipophilic drugs, whereas changed muscle mass and redistributed body water affect the distribution of hydrophilic drugs.

As shown in Table 5, in cachectic patients a reduced volume of distribution (except for carboplatin) was ascertained for both hydrophilic (ganciclovir) and lipophilic (rifabutin, saquinavir) drugs. This could be explained by the simultaneous loss of fat and lean body mass in cachectic patients which leads to reduction of space to which the drugs could distribute. Reduced volume of distribution results in higher fluctuations of drug plasma concentrations with higher peak plasma concentrations. 

Table 4 presents studies that correlated the parameters of body composition and pharmacokinetic parameters of drugs in patients with chronic disease. Volume of hydrophilic fluorouracil distribution positively correlated with amount of total body water, and lipophilic rifabutin had reduced volume of peripheral compartment in patients with cachexia, which may be due to reduced body-fat content. However, patients with reduced lean and/or fat mass are not necessarily cachectic. Lower drug clearance in patients with reduced lean body mass or body weight may simply be due to smaller volumes of liver and kidneys [47,48]. Study of Kuester et al. investigated the pharmacokinetics of monoclonal antibody matuzumab in relation to fat-free mass (FFM) and found a correlation between FFM and clearance [22]. Because these drugs follow completely different pharmacokinetics than classic small molecules, we cannot interpret these findings with classic ADME processes.

In addition to changes in body composition, altered concentrations of plasma proteins may affect the distribution of highly protein-bound drugs. Hypoalbuminemia is common in patients with cachexia [3,49,50] and could cause an increase in the unbound concentration of mostly acidic drugs (like for example warfarin, digoxin, phenytoin). An increased free fraction of a drug may lead to an increased therapeutic effect, but also to faster elimination from the body. Inversely, serum concentration of alpha-1-acid glycoprotein, an acute phase protein that mostly binds alkaline drugs (e.g., propranolol, diazepam etc.), is suggested to be increased in cachexia [51] and could therefore increase the bound fraction of such drugs. None of the studies identified in our systematic search measured the unbound fraction of the drug to observe changes at this level of pharmacokinetics.

In summary, distribution volumes of drugs appear to be reduced in cachexia. This could lead to higher fluctuations of drug plasma concentrations and higher peak concentrations in central compartment. With regard to established body composition changes in cachexia and no data about drug plasma protein-binding, studies in this field would be relevant, particularly to evaluate risk for higher than therapeutic concentrations, associated with potential side-effects. 

### Drug metabolism in cachexia

As many studies indicate, metabolism of carbohydrates, proteins, and lipids is altered in cachexia [52–55]. Although enzymes of drug metabolism differ from those involved in the metabolism of nutrients, they are also exposed to an altered cell environment and could consequently also be affected by cachexia. Malnutrition and cachexia were shown to be associated with reduced content of some cytochromes in the human liver, which could prolong drug half-life and require prolonged dosing intervals or reductions in the daily dose [56]. In addition, chronic inflammation has been shown to change the concentration of drug-metabolizing enzymes and transporters in various tissues [57].

Studies presented in Table 5 investigated drugs primarily metabolized by CYP3A4. Despite similar metabolic pathway, the influence of cachexia on plasma concentrations was divergent, although plasma concentrations of most of the drugs (oxycodone, rifabutin and saquinavir) were elevated. With oral administration, we are unable to differentiate between changes in absorption from changes in clearance and volume of distribution. The results are therefore presented as Cl/F and Vd/F (volume of distribution divided by bioavailability). However, Naito et al. [9] overcame this problem by measuring the concentration of the parent drug and its metabolite. Lower conversion of oxycodone to noroxycodone was shown in cachectic patients. 

In summary, available data suggest drug metabolism is reduced in cachexia. This may lead to higher concentrations of a parent drug or slower formation of metabolites. Therefore, in patients who developed cachexia, doses of drugs with active parent drugs may need to be reduced and doses of drugs with active metabolites may need to be increased in order to obtain the same concentrations of active moiety as prior to cachexia. Longitudinal studies comparing metabolism in chronic disease patients prior to and with established cachexia would give relevant insight into this issue. 

### Drug excretion in cachexia

There is no evidence that cachexia influences renal function, although higher incidence of new renal failure after valvular surgery was associated with cardiac cachexia [58]. The pharmacokinetics of intravenously administered carboplatin, which is eliminated only via kidneys, was not influenced by cachexia [11] and half-life of another renally excreted drug ganciclovir was also not prolonged in cachectic patients [14]. However, due to muscle mass loss in cachectic patients, renal function may be overestimated if based on serum creatinine concentration. This may result in overdosing of renally excreted drugs (e.g., carboplatin) in cachectic patients [59,60].

Renal function guides decision about therapy initiation/termination and dose adjustment, thus reliable estimation of renal function is crucial in clinical practice. For cachectic patients, it would therefore be relevant to compare different formulas for estimation of renal function with established methods (e.g. inulin or iohexol clearance) or with body composition parameters. 

### Limitations of the systematic search

The main problem encountered in this systematic search was the lack of a uniform definition of cachexia. The first consensus definition was published in 2008 [3], and even since then it has not been universally accepted. Consequently, we selected papers that included patients with chronic diseases who experienced some type of wasting or were already underweight. However, this may not be fully representative of the cachectic population.

The number of drugs studied was small and so was the assortment of chronic diseases, which makes it difficult to draw general guidelines for drug dosage adjustment in cachexia. Moreover, the results of the included studies were difficult to summarize due to their differing pharmacokinetic data presentations. 

## Conclusion

There is lack of data about the pharmacokinetics of drugs in cachexia. However, a pattern of altered absorption, reduced volume of distribution and impaired metabolism appears to be present. We were able to identify only studies that were conducted in cancer and HIV-infected patients, whereas cachexia in cardiopulmonary and other diseases still needs to be investigated. An additional search of clinical trial public repository (ClinicalTrials.gov) identified only one study with longitudinal patient assessment [61]. With an increasing burden of cachexia and better awareness about the cachexia of chronic disease, further research about drugs pharmacokinetics in body wasting and cachexia, along with evaluation of pharmacodynamics, is warranted**.**

## Supporting Information

Checklist S1 PRISMA checklist.
(DOC)
